# Supplementary material for: Antibodies against SARS-CoV-2 among health care workers in a country with low burden of COVID-19
Source: PLoS One. 2020 Dec 3;15(12):e0243025. doi: 10.1371/journal.pone.0243025 (PMC7714091; doi:10.1371/journal.pone.0243025)
Supplement: S1 Appendix — (DOCX) [file pone.0243025.s001.docx]

**Appendix: Evaluation of sensitivity and specificity of GeneBody COVID-19 IgM/IgG antibody panel**

To evaluate the sensitivity and specificity of GeneBody COVID-19 IgM/IgG antibody we used two panels. One panel included plasma from 107 hospitalized symptomatic COVID-19 patients RT-PCR positive (Panel A) and the second panel included 150 plasma samples collected during 2017-2019 (Panel B).

Panel A. 53 samples were collected 0-20 days and 54 samples 21-50 days from symptom onset; 36/53 and 47/54 were reactive respectively, for an overall sensitivity 83/107 (77.6%) (95%CI68.5, 85.1). The sensitivity according to symptom onset was, 68% (95%CI 53.7, 80.0) and 87% (95%CI 75.1, 94.6) among patients sampled 0-20 days and 21-50 days post symptoms onset, respectively. These sensitivity values are similar with an FDA approved Roche Elecsys Anti-SARS-CoV-2 assay [0-20 days: 13/18 (72%) (95%CI 46.5, 90.3); 21-50 days 64/73 (88%)(95%CI 77.9, 94.2)].^1^

Panel B.250/250 (100%) (95%CI 97.6, 100.0) scored negative by GeneBody test for a specificity 100%. Specificity by Elecsys test was 472/472 (100%) (95% CI 99.2, 100.0).^1^ The specificity of chromatographic RDT immunoassays was found remarkably high 99.5% (95%CI 98.7, 99.9) using large number of negative plasma samples collected before pandemic.^2^

Weakly positive samples by GeneBody COVID-19 IgM/IgG assay.

According to the manufacturer, the detection limit of the assay was 1.84 s/co for IgM and 1.57 s/co for IgG. Samples with s/co between 1.0 and 1.84 for IgM and between 1.0 and 1.57 are considered a grey zone or weakly positive. During the screening of hospital personnel 16 weakly positive samples were identified. All participants with weakly positive results were invited for retesting 2 weeks later. 14 individuals participated in re-testing, 13/14 were negative and 1 seroconverted to positive.

**References-Appendix**

1. Evaluation of Roche Elecsys Anti-SARS-CoV-2 serology assay for the detection of anti-SARS-CoV-2 antibodies. Public Health England. https://www.gov.uk/goverment/publications/covid-19-laboratory-evaluations -of serological-assays.
2. Erikstrup C, Hother CE, Pedersen O, Molbak K, Skov RL, Holm DK, et al. Estimation of SARS-CoV-2 infection fatality rate by real-time antibody screening of blood donors. medRxiv2020.04.24.20075291; doi: https://doi.org/10.1101/2020.04.24.20075291
