# Supplementary material for: Antibodies against SARS-CoV-2 among health care workers in a country with low burden of COVID-19
Source: PLoS One. 2020 Dec 3;15(12):e0243025. doi: 10.1371/journal.pone.0243025 (PMC7714091; doi:10.1371/journal.pone.0243025)
Supplement: Attachment — Submitted filename: Response to reviewers.docx [file pone.0243025.s002.docx]

**To the Editor of PLOS ONE**

Dear Dr. Dijkman,

Thank you for the valuable comments. To address all the concerns related to anti-SARS-CoV-2 rapid test assay we performed de-novo estimation of clinical sensitivity, clinical specificity, comparative efficacy and we used a strict quality control to accept positivity for capillary blood samples.

The clinical sensitivity and the comparative performance of GeneBody with Abbott antibody test (SARS-CoV-2 IgG – Abbott, Chicago. IL, USA) was assessed in panel A. Panel A includes 116 serum or plasma samples from 116 COVID-19 patients with clinical disease and positive RT-PCR. Both assays are based on the nucleocapsid antigen.

There was no statistical significance difference in clinical sensitivity of GeneBody and Abbott assays and their peak clinical sensitivity was similar (Table 1).

We de-novo estimated the clinical specificity of GeneBody assay on panel B, including 250 serological samples collected before COVID-19 epidemic. The specificity was found 100% , suggesting that the GeneBody assay is appropriate for studies in low prevalence populations.

Further quality controls were included. Positive capillary samples by GeneBody were tested 3 times. Two times at the date of testing and a third time 1-2 weeks later. All samples exceeding the cut-off recommended by manufacturer (1.84 s/co for IgM and 1.57 s/co for IgG) were found positive in all tests. The great majority of samples scored as weakly positive or grey zone (1.0 – 1.84 s/co for IgM and 1.0 – 1.57 s/co for IgG) were found negative when tested 1-2 weeks later (see Validation of SARS-CoV-2 antibody testing).

To assess the concordance between capillary and venous blood we tested serum samples and capillary results tested in the same day. There was 100% concordance. Finally, we adjusted the estimated prevalence for the clinical sensitivity and specificity of GeneBody assay (see Statistical analysis).

The choice of GeneBody test was based on the preliminary evaluation of a handful number of rapid tests taking advantage of our panel A and panel B. Our study was the first population study of anti-SARS-CoV-2 in Greece and later studies for anti-SARS-CoV-2 confirmed our findings.

Therefore, we believe that our results are valid and describe adequately the prevalence of anti-SARS-CoV-2 in the health care workers population in Greece.

Our detailed response in Reviewers follows:

**Reviewer #1 Comments- Suggestions:**

Suggestions for improvement

- line 111, please add the viral protein that is used as target in the GeneBody COVID-19 antibody detection test.

- line 140-141 and line 158 – 159: It is mentioned that seroprevalence is 10, 4 or 20 times higher than the general population. However, these numbers are probably not significant as the CI of the hospital tests is so wide. Please provide P-values, and mention whether there is a significant difference (or not).

**Response to Reviewer #1**

Information pertaining to viral protein used in the assays was added in line 134 of the non-tracking final manuscript: “Both assays were based on nucleocapsid protein as antigen.”

The statistical non-significance was included in lines 183 of the non-tracking final manuscript: “…although the differences were not statistically significant.”

**Reviewer #2 Comments- Suggestions:**

**Comment 1:** Pichogiou et al. perform a serological testing for COVID-19 among health care workers in Greece, a country with low burden of COVID-19. First, the major issue is lack of detailed description for the commercial kit from Genebody Inc they used for antibody testing. what is the catalogue Number? Is it a quantitative or qualitative gold immunochromatography assay? It will be great if the authors use alternative assay to confirm the positive samples. Otherwise, it is hard to know the seropositive individuals identified from this assay were really positive or false positive. Also, the author mentioned that "capillary blood" was used for testing, instead of venous blood. From our practical experience, capillary blood might cause false positivity.

**Response to Reviewer #2**

**Comment 1:** The clinical sensitivity and the comparative performance with Abbott test was assessed in panel A. Panel A includes 116 samples from 116 COVID-19 patients with clinical disease and positive RT-PCR. Both assays are based on the nucleocapsid antigen. There was no statistical significance difference in clinical sensitivity of GeneBody and Abbott assays and their peak sensitivity was similar (Table 1).

We de-novo estimated the clinical specificity of GeneBody assay which was on panel B, including 250 serological samples collected before COVID-19 epidemic. The specificity was found 100% , suggesting that the GeneBody assay is appropriate for studies in low prevalence populations.

Further quality controls were included. Positive samples in capillary blood were tested 3 times. Two times at the date of testing and a third time 1-2 weeks later. All samples exceeding the cut-off recommended by manufacturer (1.84 s/co for IgM and 1.57 s/co for IgG) were found positive. The great majority of samples scored as grey zone were found negative when tested 1-2 weeks later.

To assess the concordance between capillary and venous blood we tested serum samples and capillary results tested in the same day. There was 100% concordance.

The GeneBody assay is a qualitative lateral flow immunoassay with catalogue number COVI040. The GeneBody Inc. is one of the leaders in rapid diagnostic testing pioneered Zika virus diagnostic tests.

**Reviewer #2 Comments- Suggestions:**

**Comment 2:** Secondly, some key details of socio-demographic characteristics of HCWs in two hospitals were missing. For examples, how many individuals in hospital-1 were front line HCW (FL-HCW)? How many of them directly exposed to COVID-19 patients without proper PPE? How many seropositive HCWs used PPE? Only identifying the seroprevalence among HCWs has limited implication for preparedness of a second wave of COVID-19.

**Response to Reviewer #2**

**Comment 2:** FL-HCW and PPE use amongst FL-HCWs directly exposed to SARS-COV-2 information has been added at line 166-168 of the manuscript “FL-HCWs accounted for 73.4% (1,097/1,495) of those tested (714/898 and 383/597 for the two hospitals respectively). Participants reporting direct exposure to known cases of COVID-19 during clinical work amounted to 11.5% (82/714), out of which 95% reported full use of PPEs.”

Regarding use of PPE amongst seropositive participants, we added line 190-191 “Amongst seropositive individuals, only 9 reported full use of PPE.”
